# Subcutaneous ICD for more and transvenous ICD for few?!

**DOI:** 10.1007/s00392-022-01990-8

**Published:** 2022-02-18

**Authors:** Amr Abdin, Suleman Aktaa

**Affiliations:** 1 Klinik Für Innere Medizin III-Kardiologie, Angiologie Und Internistische Intensivmedizin, Universitätsklinikum Des Saarlandes, Kirrberger Strasse 100, 66421 Homburg, Germany; 2 Leeds Institute for Data Analytics, University of Leeds, Leeds, UK; 3 Leeds Institute of Cardiovascular and Metabolic Medicine, University of Leeds, Leeds, UK; 4 Department of Cardiology, Leeds Teaching Hospitals NHS Trust, Leeds, UK

**Keywords:** Sudden cardiac death, Implantable cardioverter defibrillator, Subcutaneous ICD

## Abstract

Implantable cardioverter defibrillators (ICDs) have been shown to reduce the risk of sudden cardiac death in primary or secondary prevention with thousands of ICDs implanted every year worldwide. Whilst ICD are more commonly implanted transvenously (TV), this approach carries high risk of peri- and post-procedural complications. Subcutaneous ICD (S-ICD) have been introduced to overcome the intravascular complications of TV system by placing all metalware outside the chest cavity for those with an indication for a defibrillator and no pacing requirements. In conclusion, a review of the current guidelines recommendations regarding S-ICD may be needed considering the emerging evidence which shows high efficacy and safety with contemporary devices and programming algorithms. A stronger recommendation may be developed for selective patients who have an indication for single-chamber ICD in the absence of negative screening, recurrent monomorphic ventricular tachycardia, cardiac resynchronization therapy, or pacemaker indication. These criteria encapsulate a large proportion (around 70%!) of all ICD eligible patients.

Despite the advances in diagnostics and therapies of cardiovascular disease, sudden cardiac death (SCD) remains a major challenge accounting for approximately 0.1–0.2% deaths per year [1]. Implantable cardioverter defibrillators (ICDs) have been shown to reduce the risk of SCD in primary or secondary prevention [1, 2] with thousands of ICDs implanted every year worldwide [3, 4]. Whilst ICD are more commonly implanted transvenously (TV), this approach carries high risk of peri- and post-procedural complications [3, 5]. For instance, pneumothorax as well as cardiac and vascular damage are recognized complications that are associated with TV-ICDs at the time of implantation (up to 3.5%) [6]. After having implanted the TV-ICD system without any acute complications, the long-term complications are faced, which are mainly lead related problems and might be seen in around 25% of TV-ICD over the first decade following implantation [3, 5, 7]. Furthermore, TV-ICD is prone to systemic infections which may result in the need for device extraction and the subsequent morbidity and mortality [8]. For illustration, mortality following TV-ICD system removal due to pocket and endovascular infection can reach 31% at 1 year [8].

Subcutaneous ICD (S-ICD) have been introduced to overcome the intravascular complications of TV systems by placing all metalware outside the thorax cavity for those with an indication for a defibrillator and no pacing requirements [3]. Pooled data from long-term registries showed the device-related complications after S-ICD implantation occurred in 11.1% of patients at 3 years [9]. Moreover, lead failures rate was rarely reported [3, 9]. However, On December 2020, a medical advisory letter regarding EMBLEM S-ICD electrode model 3501 (Boston Scientific, Marlborough, MA) was released by Boston Scientific Corporation [10]. According to their data, there have been 27 cases of electrode body fractures, resulting in life-threatening events of 1 in 25,000 at 10 years. The overall lead failure rate is still lower than what has been reported with TV-ICD leads [5, 11, 12].

Notwithstanding that international guideline, including these of the European Society of Cardiology (ESC) and the American College of Cardiology (ACC) / American Heart Association (AHA) recommend S-ICD only as an alternative strategy to TV-ICD for eligible patients [1, 2], their recommendations were based mainly on early S-ICD studies which were observational prospective analyses [3]. These studies could prove the reliability of S-ICD in detecting and terminating ventricular arrhythmias [3]. However, more recently, the PRAETORIAN (Prospective, Randomized comparison of subcutaneous and transvenous Implantable cardioverter defibrillator therapy) [13] and the Understanding Outcomes With the S-ICD in Primary Prevention Patients With Low Ejection Fraction (UNTOUCHED) trials [14] were published. The PRAETORIAN trial was a randomized trial which included 849 patients with an indication for ICD therapy to either TV- or S-ICD. After a median follow-up of 48 months, the primary endpoint of inappropriate shocks or device-related complications occurred in 15.7% in TV-ICD and 15.1% in S-ICD recipients (*P* = 0.01 for noninferiority) [13]. Interestingly, during 4 years of follow-up, only 0.9% of patients required TV-ICD for pacing and 0.2% for ATP.

The UNTOUCHED study enrolled 1111 patients with a primary prevention ICD indication, and no indication for pacemaker [14]. The inappropriate shock-free rate at 18 months (i.e., the study’s primary endpoint) was 95.9% (*P* < 0.001 vs the prespecified performance goal of 91.6%). During 18 months of follow-up, only 0.2% of patients required TV-ICD for ATP.

Hence, with increasing physician experience in implantation and programming as well as improvements in the detection, filter, and discrimination algorithms, the rate of inappropriate shocks has decreased over the years. Indeed, the yearly rate of inappropriate shocks observed in UNTOUCHED patients was 3.1%, which was further reduced to 2.4% with the Gen 3 devices. This rate was with the range of that observed in current TV-ICDs trials [12, 14].

In view of this:

## Novel perspectives

Why not S-ICD as a first line therapy for all ICD patients?!

According to the Italian S-ICD survey around 88% of all ICD indicated patients were eligible to receive an S-ICD [11]. However, S-ICD was only implanted in 12% of patients with no cardiac synchronization therapy (CRT) indication. Although the most common reasons for preferring a TV-ICD over an S-ICD were the need for permanent pacing or ATP therapy, at the time of ICD implantation, only 7–10% of patients fulfilled conditions for Class I recommendation for permanent pacing [15]. An additional 4% of patients presented with a history of unstable monomorphic ventricular tachycardia that might have been treatable with ATP. Furthermore, even in countries with high ICDs implantation rates such as Germany, only 6% of the 23,000 patients who had ICD implantation in 2018 received S-ICD [4].

The modular cardiac rhythm management (mCRM) system, involving of a communicating ATP-enabled EMPOWERTM LCP and EMBLEMTM platform S-ICD system, which enables the coordination of leadless pacing and defibrillator therapy delivery [16]. The modular ATP is a current randomized controlled trial assessing the safety and efficacy of an individualized approach to ICD therapy that providing a solution for patients who may develop a need for pacing or ATP in the future [16].

**Fig. 1:**
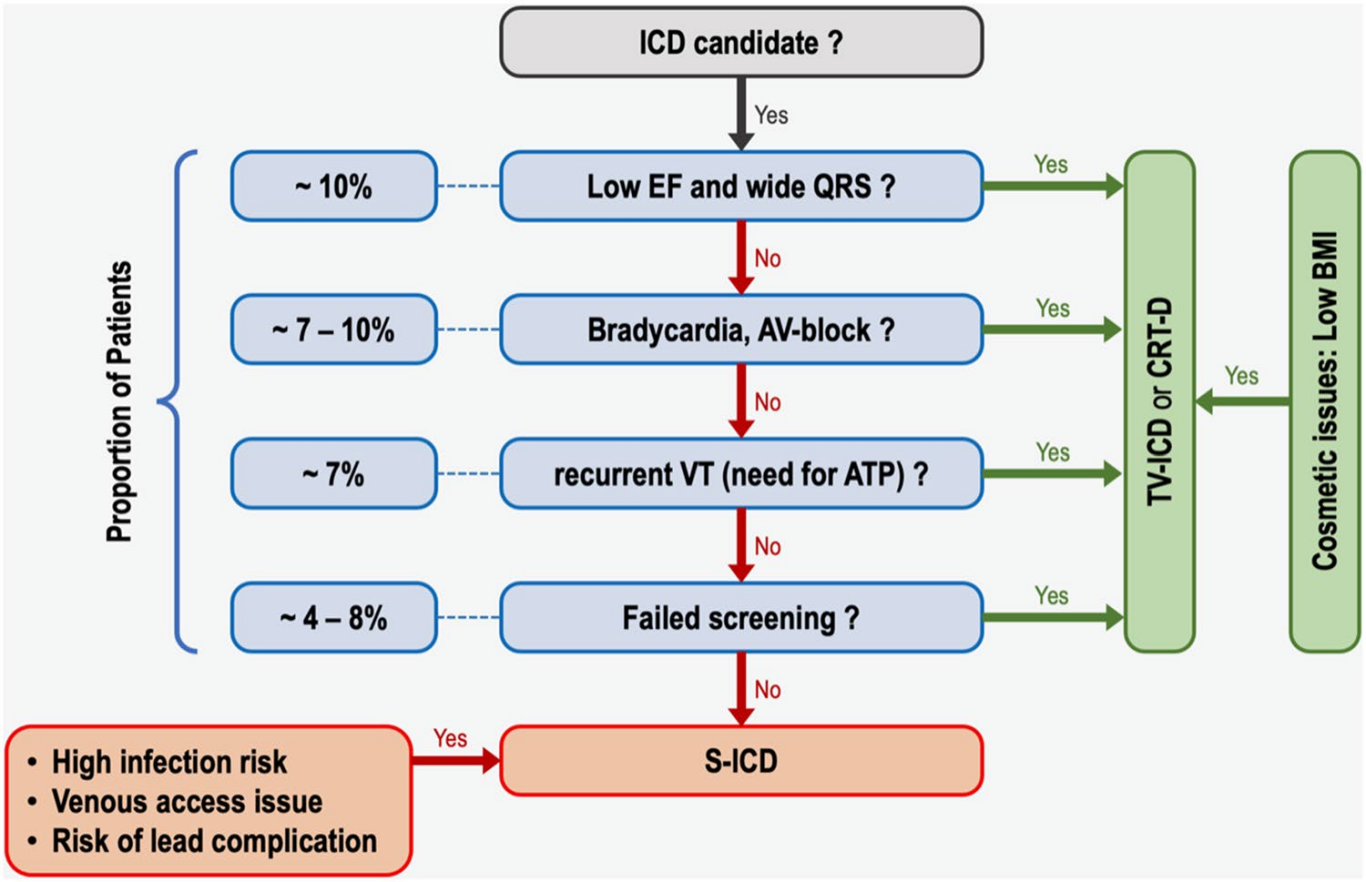
Algorithm to define suitable patients for S-ICD. This figure shows an algorithm to define suitable patients for S-ICD. Patients with low left ventricular ejection fraction (EF) and wide QRS complex (accounting 10% of all ICD population at implant) should receive a CRT-D. Patients with an indication for pacemaker should receive a TV-ICD or CRT-D (accounting 7–10% of all ICD population at implant). Patients with recurrent monomorphic ventricular tachycardia (VT) should also receive a TV-ICD (7% of all ICD population at implant). After excluding the above-mentioned group of patients a screening for S-ICD should be performed (can be failed in 4–10%), Data taken from [15–19]

In conclusion, a review of the current guidelines recommendations regarding S-ICD may be needed considering the emerging evidence which shows high efficacy and safety with contemporary devices and programming algorithms. A stronger recommendation may be developed for selective patients who have an indication for single-chamber ICD in the absence of negative screening, recurrent monomorphic ventricular tachycardia, cardiac resynchronization therapy, or pacemaker indication. According to the existing evidence, these criteria encapsulate a large proportion (around 60%!) of all ICD eligible patients (Fig. 1) [15–19].

Other criteria which may favor S-ICD include, young patients with a long-life expectancy and patients at risk of infection with diseases such as diabetes mellitus or renal insufficiency requiring dialysis or venous access issues should be also provided with an S-ICD as far as possible in order to avoid complications and to preserves the venous system for other purposes [11, 14].

Nonetheless, S-ICD has additional important limitations which are the large pulse generator, with anticipated life battery shorter than TV-ICD and the relatively high costs [14]. Moreover, the current recommendation for S-ICD implantation is to perform defibrillation testing (DFT) to determine the ability of the device at terminating ventricular fibrillation [12]. In a recent published propensity-matched study, DFT was not associated with significant differences in ineffective shocks and cardiovascular mortality. The PRAETORIAN-DFT is a current randomized controlled trial assessing DFT in the S-ICD which should elucidate the need for DFT in the S-ICD [20].

Based on the above, appropriate patient selection with risk–benefit assessment alongside contemporary device programming is an essential parameter to the selection and success of S-ICDs.
